# Study protocol of the Healthy High School study: a school-based intervention to improve well-being among high school students in Denmark

**DOI:** 10.1186/s12889-020-8194-y

**Published:** 2020-01-22

**Authors:** Camilla Thørring Bonnesen, Mette Toftager, Katrine Rich Madsen, Stine Kjær Wehner, Marie Pil Jensen, Johanne Aviaja Rosing, Bjarne Laursen, Naja Hulvej Rod, Pernille Due, Rikke Fredenslund Krølner

**Affiliations:** 1 Centre for Intervention Research in Health Promotion and Disease Prevention, National Institute of Public Health, University of Southern Denmark, Studiestræde 6, 1455 Copenhagen, Denmark; 2 Section of Epidemiology, Department of Public Health, University of Copenhagen, Bartholinsgade 6Q, 1356 Copenhagen, Denmark

**Keywords:** Well-being, Stress, Sleep, Physical activity, Sedentary behaviour, Meal habits, Sense of community, Randomised controlled trial, High school, Study design

## Abstract

**Background:**

The prevalence of low well-being, perceived stress and unhealthy behaviours is high among high school students, but few interventions have addressed these problems. The aim of this paper is to present a study protocol of a cluster randomised controlled trial evaluating the Healthy High School (HHS) intervention programme. The intervention programme is designed to improve well-being (primary outcome) by preventing 1) stress and promoting 2) sleep, 3) sense of community, 4) physical activity (PA) and 5) regular and healthy meals among high school students in Denmark.

**Methods:**

The development of the HHS study was guided by the Intervention Mapping protocol. The intervention comprises four components: 1) a teaching material, 2) a smartphone app, 3) a catalogue focusing on environmental changes, and 4) a peer-led innovation workshop aiming at inspiring students to initiate and participate in various movement activities. The HHS study employs a cluster-randomised controlled trial design. Thirty-one high schools across Denmark were randomly allocated to intervention (16 schools) or control (15 schools) groups. The study included all first-year students (~ 16 years of age) (*n* = 5976 students). Timeline: Intervention: August 2016 – June 2017. Collection of questionnaire data: Baseline (August 2016), 1st follow-up (May 2017) and 2nd follow-up (April 2018). All students were invited to participate in a monthly sub-study about perceived stress using text messages for data collection (September 2016 – June 2017). PA was objectively assessed among a sub-sample of students using accelerometers (Axivity, AX3) in August 2016 and May 2017. Primary outcome measures: Student well-being measured by the Cantril Ladder and the five item World Health Organisation Well-being Index (individual level outcomes). Secondary outcome measures: Stress (10-item Perceived Stress Scale), sleep (quantity and quality), PA (hours of moderate-to-vigorous PA per week, hours of daily sedentary time and average daily PA), meal habits (daily intake of breakfast, lunch, snacks and water), and strong sense of community in class and at school, respectively (individual level outcomes). The study encompasses process and effect evaluation as well as health economic analyses.

**Trial registration:**

ISRCTN ISRCTN43284296, 28 April 2017, retrospectively registered.

## Background

The Danish National Youth Study 2014 (DNYS) shows that most high school students are satisfied with their lives and perceive their health as good. However, remarkable percentages report mental health problems such as low life satisfaction (29%), high levels of stress (12%) and loneliness (9%) [1]. Health-related behaviours that underlie the major non-communicable diseases are usually initiated or reinforced during adolescence [2], and infrequent meal consumption [1], an inactive lifestyle [1, 3, 4] and inadequate sleep [1, 5–7] are common among adolescents in Denmark and other high income countries. Health-related behaviours established during youth tend to track across lifespan and adversely affect health in adulthood [8–10]. Conversely, previous research indicates that adoption of a healthy lifestyle in youth can have protective effects against the onset of chronic diseases [11].

There is a strong rationale for intervening among high school students (15–18 years of age): the late adolescent period is characterised by change of behavioural focus and influence in which peers and school environments become increasingly important and parental influence and control diminish [12, 13]. This period may thus be a window of susceptibility for changing behaviours, and the school environment can be a strong player and a possible supporter for the development of healthy behavioural patterns in youth [14]. However, the majority of school-based health promotion interventions have focused on children in primary school, and only few interventions have targeted older adolescents including high school students [15].

Previous health promoting interventions have mostly targeted single health behaviours, but evidence indicates that health behaviours among adolescents are interrelated and form behavioural clusters [3, 16–21]. Furthermore, intrapersonal determinants regarding these behaviours are likely to cluster [17], and a positive change in intrapersonal determinants of one behaviour might, therefore, induce a similar change in a related behavioural determinant [21, 22]. This has prompted the development of multiple behaviour change interventions [21]. A recent systematic review concluded that whole-school-based interventions targeting multiple adolescent behaviours simultaneously appear to be the most effective way to promote healthy behaviours among adolescents [23]. Previous intervention studies among older adolescents have been limited by lack of theoretical frameworks to guide intervention planning and by methodological weaknesses [15, 24] such as small homogenous samples, lack of comparison groups [15], and short-term post-intervention measures [25]. Further, most previous studies have not included outcome measures related to all program components limiting the understanding of how each of the intervention components worked and what may mediate the effects of the intervention [15, 24, 25]. Finally, few studies have included fidelity measures, limiting the understanding of any association between dose and response and whether the interventions were implemented as intended [15, 25].

### Aim

The overall aim of the Healthy High School (HHS) study is to develop, implement and evaluate a theory- and evidence-based, multi-component school-based intervention to improve well-being (primary outcome) by preventing 1) stress and promoting 2) sleep, 3) sense of community, 4) physical activity (PA) and 5) regular and healthy meals among high school students in Denmark. This paper presents the study protocol of the HHS intervention.

### Specific objectives

The primary objective is to assess the effectiveness of the HHS intervention by comparing subjective well-being in intervention and control schools. Study hypothesis: at 9-month follow-up there will be a 6%-point difference in the prevalence of students with a high level of life-satisfaction in intervention schools compared to control schools. This difference will be sustained at 20-month follow-up.

Secondary objectives are to compare differences in the following outcomes between the two groups at 9-month and 20-month of follow-up: Perceived stress, sleep quantity and quality, PA, meal habits, and sense of community within the school class and at school. Additional objectives are to conduct a mixed-methods process evaluation and a health economic evaluation.

## Methods and design

### Study design

A cluster-randomised controlled design is used for the effect evaluation of the intervention with baseline measurements in the beginning of the school year (August 2016), and follow-up measurements in the end of the school year (May 2017) and one year after the end of the intervention (April 2018).

### Setting and target group: the upper secondary school leaving examination (STX)

Education in Denmark is financed by taxes and, therefore, free of charge at all levels. The Danish upper secondary education system divides into two branches: 1) *vocational upper secondary education and training programmes* qualifying primarily for access to the labour market, and 2) *general upper secondary education programmes* qualifying for access to higher education. General upper secondary education comprises four academically orientated education programmes: the upper secondary school leaving examination (STX), the higher preparatory examination (HF) (both referred to as high school), the higher commercial examination (HHX), and the higher technical examination (HTX) [26]. The HHS intervention is designed to STX, but HF classes are invited if they are offered at the same school as a participating STX (an inclusive approach). Due to the optional set-up and participation of HF classes at each school, the data from HF students will not be included in the main effect analysis of the study. The rest of the paper will only concern STX.

Danish high schools are uniformly organised with a standardised school class structure and once-a-year enrolment [27]. The high schools adhere to the regulations issued by The Danish Ministry of Education and are self-governing independent institutions. The school board has the overall responsibility for running and managing the educational and teaching activities as well as the administration and financial management of the high schools. Most members come from outside the high school, mainly from the local area. As a group, the members of the board should have competencies that will contribute to promoting the high schools current and future activities. The teachers and students each appoint two representatives to the school board [28]. Students also have the opportunity to influence their high school by forming student councils [29].

### Recruitment

**Fig. 1:**
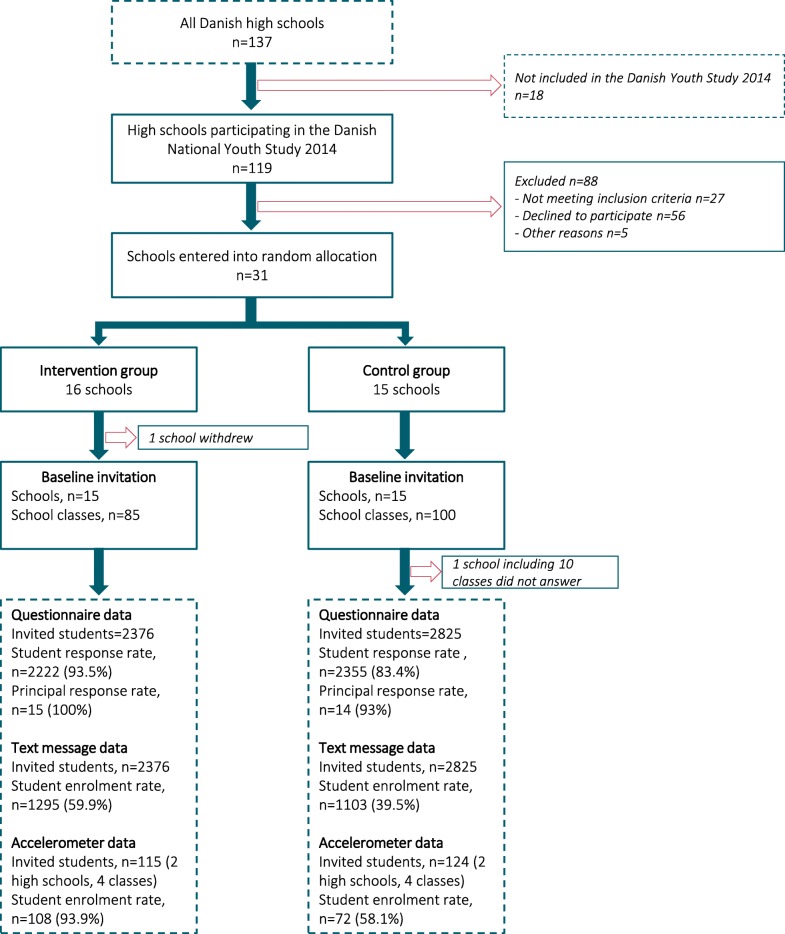
Flow diagram of recruitment, randomisation and participation in the Healthy High School study

Figure 1 shows the flow of the sampling process. We wanted to recruit high schools with the lowest proportion of students with a high level of well-being to reach those in most need (strategic sample). We ranked the 119 high schools that participated in the DNYS [30] based on the proportion of students with a high level of life satisfaction (step 9–10 on the Cantril Ladder of Life Scale. See description below). Eligible high schools had minimum two school classes of first-year students. Exclusion criteria were: students attending International upper secondary school classes, students in classes participating in the Team Denmark programme (educational opportunities for elite athletes), high schools where the majority of students live on the premises (boarding schools) (*n* = 3), and high schools that were invited to participate in another study at The National Institute of Public Health, University of Southern Denmark, in the same period (*n* = 24).

Initially, we invited 40 high schools by telephone. Immediately after the telephone conversation, high schools received recruitment material for the school management, teachers and student council. The following weeks, the high schools were contacted again to follow-up on the invitation. Due to a challenging recruitment process we had to give up our strategic sampling strategy and we invited all high schools participating in the DNYS which adhered to our exclusion and inclusion criteria (*n* = 92). Thirty-one of the 92 eligible high schools agreed to participate, 56 high schools declined, and five high schools did not revert before randomisation. Fifteen of the 31 participating high schools were from the first recruitment round.

### Randomisation

The 31 recruited high schools were randomly allocated into the intervention (*n* = 16) or control groups (*n* = 15) by computer-based random number generation. After randomisation, one high school withdrew from the study leaving 15 intervention high schools and 15 control high schools in the final study (Fig. 1).

### Control group

The control schools participated in the data collection, received no intervention and were encouraged to continue as originally planned before being contacted by the research group. The control schools will get access to the intervention material after the study period.

### Conceptual framework and planning model

The HHS study builds on a socio-ecological framework which acknowledges that individual behaviour such as PA or meal habits is shaped by factors at multiple levels [31]. Ecological models emphasise the structural, physical and political context while incorporating social and psychological influences. A practical implication of the ecological framework is multi-level interventions which use multiple strategies to behaviour change. The Intervention Mapping protocol (IM) is used to plan the intervention, implementation and evaluation of the HHS study in a systematic fashion. The IM proposes six consecutive steps for the development of health promotion programmes based on theory, best available evidence and additional qualitative and quantitative research. The six steps included in the IM are: 1) conducting a  needs assessment, 2) preparing matrices of change objectives, 3) selecting theory-informed intervention methods and practical applications, 4) producing program components and materials, 5) planning program adoption, implementation, and sustainability, and 6) evaluation planning. As suggested by the authors of the IM, the planning process was conducted in an iterative fashion, moving backward and forward between the steps and tasks [32]. A detailed description of the development of the HHS intervention using the IM steps is described in a separate paper (Bonnesen et al., in preparation).

### Programme theory

The aim of the HHS intervention is to promote well-being among high school students (primary outcome) by providing them with energy for the school day. To achieve this goal, the intervention is designed to prevent 1) stress and promote 2) sleep, 3) sense of community, 4) PA and 5) regular and healthy meals (secondary outcomes). Table 1 shows an overview of the formulated program objectives, outcomes and performance objectives of each level of the intervention.

**Table 1:** Overview of formulated program objectives, outcomes and performance objectives for each level of the intervention

PROGRAM OBJECTIVE	Behavioural outcomes –individual level Performance objectives	Environmental outcomes – organisational level: School management Performance objectives	Environmental outcomes – organisational level: School staff Performance objectives
1. Prevention of chronic stress among high school students in Denmark	1.1. Students prevent chronic stress 1.1.1. Students are able to avoid potential stressors 1.1.2. Students are able to manage unavoidable stressors 1.1.3. Students are able to recognise early warning signs and symptoms of stress	1.2. The school management create a school environment that prevent chronic stress among students 1.2.1. The school management allocates resources for teachers to plan and coordinate the students’ homework and assignments 1.2.2. The school management allocates resources for student counsellors to introduce students to time management tools 1.2.3. The school management allocates resources for student counsellors to monitor the overall well-being of the students 1.2.4. The school management allocates resources for homework assistance 1.2.5. The school management recommends teachers to reduce the number of grades 1.2.6. The school management recommends teachers to reduce the number of new assignments 1.2.7. The school management allocates resources for mindfulness courses 1.2.8. The school management allocates resources for teachers to implement the stress teaching materials 1.2.9. The school management improves the physical environment 1.2.10. The school management formulate a stress policy	1.3. School staff create a school environment that prevent chronic stress among students 1.3.1. Teachers plan and coordinate the students’ homework and assignments 1.3.2. Student counsellors introduce students to time management tools 1.3.3. Student counsellors monitor the overall well-being of the students 1.3.4. Teachers (and second- or third-year students) organise homework assistance 1.3.5. Teachers reduce the number of grades 1.3.6. Teachers reduce the number of new assignments 1.3.7. Teachers organise mindfulness courses 1.3.8. Teachers implement the stress teaching materials
2. Promotion of healthy sleep habits among high school students in Denmark	2.1. Students improve their sleep habits (sleep quantity and sleep quality) 2.1.1. Students are able to go to bed earlier at night (sleep quantity) 2.1.2. Students are able to fall asleep at night (sleep quality) 2.1.3. Students are able to sleep better at night (sleep quality)	2.2. The school management creates a school environment that promote healthy sleep habits among students 2.2.1. The school management introduces a rule for assignment deadlines 2.2.2. The school management allocates resources for teachers to implement the sleep teaching materials 2.2.3. The school management formulate a sleep policy	2.3. School staff create a school environment that promote healthy sleep habits among students 2.3.1. Teachers implement a rule for assignment deadlines 2.3.2. Teachers implement the sleep teaching materials
3. Increasing PA and reducing sedentary time in weekdays among high school students (before/during/after school hours) in Denmark	3.1. Students are more physically active and spend less time on sedentary activities 3.1.1. Students particiapte in MI in the classroom 3.1.2. Students are physically active during recess 3.1.3. Students are physically active during Physical Education and Sport 3.1.4. Students develop and implement new PA initiatives 3.1.5. Students participate in PA initiatives before/after school hours	3.2. The school management create a school environment that promotes PA before, during and after the school day among students 3.2.1. The school management allocates resources for increasing the number of PA initiatives before/after school hours including support for the implementation of PA initiatives derived from the Young & Active workshop 3.2.2. The school management allocates resources for MI in the classroom 3.2.3. The school management allocates resources for teachers to implement the PA teaching materials 3.2.4. The school management allocates resources for providing more PA opportunities during recess 3.2.5. The school management formulate a PA policy	3.3. School staff creates a school environment that promotes PA before, during and after the school day among students 3.3.1. Teachers implement MI in the classroom 3.3.2. Teachers implement the PA teaching materials 3.3.3 Teachers (and second- and third year students) facilitate the Young & Active workshop where students develop new PA initiatives 3.3.4. Teachers (and second- and third year students) support first year students in the implementation of new PA initiatives after the Young & Active workshop
4. Promoting regular meal habits and increasing intake of healthy snacks and water among high school students in Denmark	4.1. Students establish regular meal habits and increase intake of healthy snacks and water (and decrease intake of unhealthy alternatives) 4.1.1. Students drink water instead of sugar-sweetened beverages 4.1.2. Students eat healthy snacks instead of unhealthy snacks 4.1.3. Students eat healthy breakfast every day 4.1.4. Students eat healthy lunch every day	4.2. The school management create a school environment that support regular meal habits and intake of healthy snacks and water 4.2.1. The school management ensures that students have access to fresh drinking water 4.2.2. The school management allocates resources for teachers to implement the healthy eating teaching materials 4.2.3. The school management meets with a professional canteen consultant and supports and encourages canteen staff to adjust their food selection 4.2.4. The school management formulate a healthy food policy	4.3. School staff create a school environment that support regular meal habits and intake of healthy snacks and water 4.3.1. School caretakers and/or canteen staff provide fresh drinking water 4.3.2. Teachers implement the healthy eating teaching materials 4.3.3. Canteen staff meet with a professional canteen consultant and adjust their food selection according to this

*PA* physical activity, *MI* movement integration

**Fig. 2:**
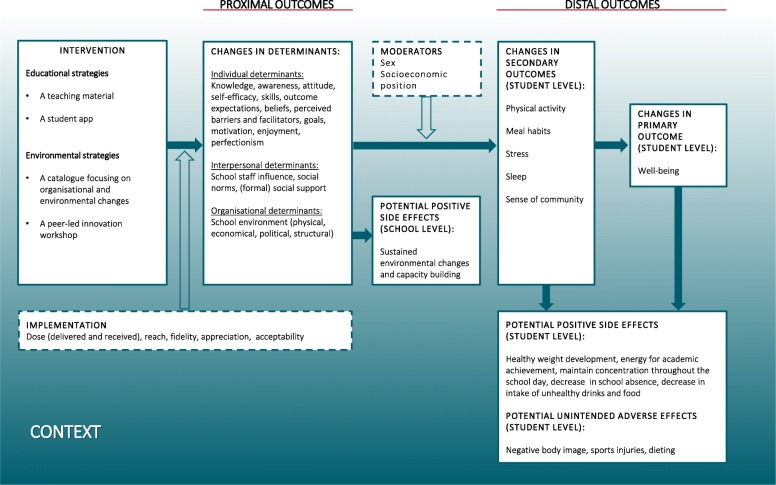
Programme Theory of the Healthy High School study

The programme theory (Fig. 2) outlines the causal chain which explains how the intervention is expected to impact well-being through changes in secondary outcomes (distal outcomes) and determinants (proximal outcomes).

The intervention strategies tailor important and changeable determinants of the secondary outcomes identified during the needs assessment including individual determinants (knowledge, awareness, attitudes, self-efficacy, skills and outcome expectations), interpersonal determinants (peer and school staff influence, social norms and social support) and organisational determinants (physical, financial, political and structural) [33–43]. The HHS study place a strong focus on creating a supportive school environment: To be able to act on e.g. acquired knowledge and skills resulting from the HHS educational strategies, students must be provided with opportunities for making healthy choices. Furthermore, we attach great importance to strong peer relations and sense of community which is reflected in three of four intervention components. The programme theory also illustrates potential unintended adverse effects as well as anticipated positive side effects of the intervention.

### Intervention

The HHS study is organized within two settings (high school and leisure time) and includes educational and environmental strategies. The HHS intervention consists of four main intervention components: 1) a catalogue focusing on organisational and environmental changes (setting: high school), 2) a teaching material (setting: high school), 3) a peer-led innovation workshop (setting: high school and leisure time), and 4) a smartphone app (setting: leisure time).

The catalogue aims to create a healthy school environment to support student behaviour change, sense of community and stress prevention either through organisational changes, e.g. a health and well-being policy, or educational initiatives (which were not subject specific), e.g. a two-hour time management course. The catalogue includes nine mandatory and seven optional initiatives and are available online for all intervention schools. The catalogue is addressed to student councils, school managements, teachers, student counsellors, canteen staff, and school caretakers.

The teaching material is designed to change social norms and cognitive factors such as knowledge, skills, attitudes, awareness and outcome expectancies through curricular activities in four subjects (Danish, Social Studies, PE and Introduction to Natural Science). It comprises 17 lessons (1440 min) and optional lessons within a Multi-Subject Coursework (one school week). Two of the environmental initiatives are integrated into each lesson: 1) movement integration (MI) in the classroom to promote PA and reduce time spent sedentarily and 2) a buddy programme for e.g. group work in class to increase sense of community. A proposal for timing of each lesson across the school-year are provided to ensure that students are exposed to the HHS material on a regular basis. Some lessons are designed to be implemented at a specific time during the year, e.g. in the first week of high school as part of introductory activities, and other lessons are designed as a collection of consecutive lessons.

The aim of the HHS app is to support and promote healthy habits and well-being outside school hours by influencing students’ knowledge, attitudes awareness, outcome expectations, and skills of four main themes; stress, sleep, PA and meal habits. The app includes articles, tracking options, recipes, tests and an option to sign up for an eight-week course on how to prevent stress, increase PA, and improve sleep or meal habits.

The Young & Active concept aims to create an inclusive and engaging PA norm at the high school that appeals to all students. The concept is based on user driven innovation methods, the Creative Platform [44], student co-determination, and a peer-led approach. The concept mainly consists of a six-step innovation workshop facilitated by university students in Sports and Health. The workshop aims to inspire high school students to invent, initiate and participate in a broad range of activities tailored to their high school which focus on increasing PA, sense of community and well-being. The university students are expected to function as role models and, thereby, increase the students’ motivation to be active and to engage in influencing the options for movement and PA at their school. To facilitate and promote establishment of new activities, students could apply for economic support from the research group (up to 40.000 DKK, equalling approximately 4800 GBP per high school) by completing a standardised grant application form.

**Fig. 3:**
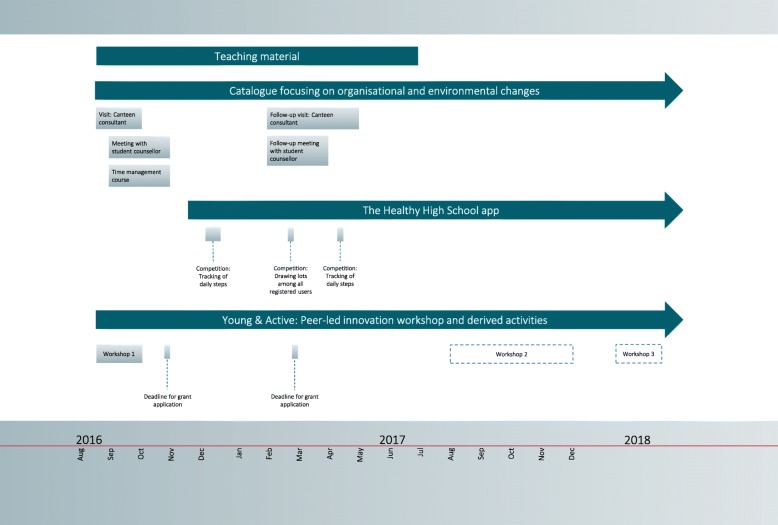
Timeline of the Healthy High School intervention

Figure 3 illustrates the overall timing of the four intervention components. Single elements of the intervention components are proposed to be implemented at specific time points emphasised in the grey boxes. The development of the intervention components and strategies are described in detail elsewhere (Bonnesen et al., in preparation).

### Project name and visual identity

The study is registered as the HHS study in the trial registry. In Danish, the project is named *en go’ Bgym’* - a wordplay meaning ‘a good start in high school’. We chose a project name with a humorous tone to appeal to young people without focusing on health. The pictorial material is designed to express situations from everyday life that students can relate to. ‘Real’ students are used instead of professional models to represent different types of students. The project name and visual identity was developed in close collaboration with a professional graphic designer.

### Implementation

The implementation of the HHS intervention was initiated in the school year 2016/17. Prior to the start of the intervention, we asked principals at all intervention schools to recruit two school coordinators; one among students and one among school staff (e.g. a teacher or the head of training division). Their tasks were to receive and redistribute information about the HHS study from the research group to teachers and students and to work as local project ambassadors. Implementation was launched by a one-day kick-off conference in the beginning of the school year 2016/17. The school coordinators and up to three other school representatives (e.g. teachers, student counsellors, canteen staff and student councils) were invited to participate. The kick-off conference was opened by a famous Danish brain researcher giving a keynote speech on the adolescent brain in relation to the main themes of the intervention. Furthermore, the research group gave a comprehensive presentation of the main goals and rationale of the intervention, an introduction to the intervention components and an overview of the evaluation design of the study. The participants experienced some of the intervention components during the conference. They met the canteen consultant who was going to visit all high schools during the intervention  period  (mandatory environmental change  initiative). They were encouraged to discuss how to implement selected interventions components during four different workshops; one that focused on the Young & Active initiative, and three that focused on the stress and sleep related initiatives. Implementation manuals for all intervention components have been developed to accompany this complex intervention. The planning of implementation and sustainability will be described in detail in a separate paper (Bonnesen et al., in preparation).

### Data collection

The following data was collected for the effect evaluation of the HHS study: 1) self-reported questionnaires from students and school administrators, 2) registration of students’ perceived stress by text messages, and 3) objective assessment of PA by accelerometer measures. All data were collected simultaneously at the intervention and control schools.

#### Student questionnaires

The student questionnaire is mainly based on validated measures from the DNYS [30] and the Health Behaviour in School-aged Children (HBSC) study [45] and supplemented with items from other studies including the Boost study [46] and the Pro Children study [47]. The items were either transferred directly or adjusted according to our study population. New items were developed specifically related to the HHS study. The questionnaire covers the following themes: socio-demographics, well-being, stress, sleep, sense of community, PA, eating and meal habits, social relations, school life, potential determinants of outcome measures, implementation measures, positive and negative side effects. The questionnaire was pre-tested among the research group and colleagues to assess time range for answering and phrasing of the questions, resulting in slight revision. New items that were related to implementation of the study and developed for the follow-up questionnaires were pilot tested on 10 students from one intervention school and revised according to observations and the students’ comments. Table 2 summarises the quantitative measures included in the HHS surveys.

**Table 2:** Quantitative data collection of the Healthy High School study

Key measures	Operationalization	Instrument	Timing of collection^*^	Data source
PRIMARY OUTCOME				
Well-being	WHO-Five Well-Being Index, Cantril Ladder of Life Scale	Web-based questionnaire	Baseline, 1st follow-up and 2nd follow-up	Students
SECONDARY OUTCOMES				
Stress	10-item Perceived Stress Scale Stress intensity, stress frequency	Web-based questionnaire Web-based questionnaire;text-messages	Baseline, 1st follow-up and 2nd follow-up Baseline, 1st follow-up and 2nd follow-up; once a month from September 2016–June 2017	Students Students; subsample of students
Sleep	Sleep quantity, sleep quality	Web-based questionnaire	Baseline, 1st follow-up and 2nd follow-up	Students
PA	Hours of moderate-to-vigorous PA per week Average daily PA	Web-based questionnaire Accelerometer	Baseline, 1st follow-up and 2nd follow-up Baseline, 1st follow-up	Students Subsample of students
Sedentary behaviour	Hours of daily sedentary time	Web-based questionnaire; accelerometer	Baseline, 1st follow-up and 2nd follow-up; baseline, 1st follow-up	Students; subsample of students
Meal habits	Daily intake of breakfast, lunch and water	Web-based questionnaire	Baseline, 1st follow-up and 2nd follow-up	Students
Sense of community	High school sense of community School class sense of community	Web-based questionnaire Web-based questionnaire	Baseline, 1st follow-up and 2nd follow-up 1st follow-up and 2nd follow-up	Students Students
PROXIMAL OUTCOMES				
INDIVIDUAL DETERMINANTS^**^				
Knowledge	Knowledge of stress, national dietary guidelines, PA guidelines and sleep recommendations	Web-based questionnaire	1st follow-up	Students
Awareness	Awareness of whether they meet national dietary guidelines, PA guidelines and sleep recommendations, and of own stress symptoms	Web-based questionnaire	Baseline, 1st follow-up and 2nd follow-up	Students
Self-efficacy	General self-efficacy	Web-based questionnaire	Baseline, 1st follow-up and 2nd follow-up	Students
Outcome expectations	Perceived effects of PA, sleep, eating breakfast/lunch, e.g. more energy, stress relief, better sleep	Web-based questionnaire	Baseline, 1st follow-up	Students
Skills	Time management skills, stress management	Web-based questionnaire	Baseline, 1st follow-up	Students
Perceived barriers and facilitators	Barriers and motivation for PA and eating regular meals	Web-based questionnaire	Baseline, 1st follow-up	Students
Perfectionism	Concern over mistakes, personal standards	Web-based questionnaire	Baseline, 1st follow-up	Students
INTERPERSONAL DETERMINANTS				
Peer- and school staff influence	Social norms, social support	Web-based questionnaire	Baseline, 1st follow-up and 2nd follow-up	Students, school administrators
ORGANISATIONAL DETERMINANTS				
Environmental conditions at school and local community	Physical, economical, political and structural	Web-based questionnaire	Baseline, 1st follow-up and 2nd follow-up	School administrators
POTENTIAL POSITIVE SIDE EFFECTS				
Healthy weight development	Body mass index determined from weight and height	Web-based questionnaire	Baseline, 1st follow-up and 2nd follow-up	Students
Energy for academic achievement	Maintenance of energy during the school day	Web-based questionnaire	Baseline, 1st follow-up	Students
Maintain concentration throughout the school day	Morning and afternoon concentration	Web-based questionnaire	Baseline, 1st follow-up	Students
Decrease in school absence	The number of school days missed due to absence the past 30 days	Web-based questionnaire	2nd follow-up	Students
Decrease in intake of unhealthy food & drinks	Intake of soft drinks, energy drinks and unhealthy snacks	Web-based questionnaire	Baseline, 1st follow-up and 2nd follow-up	Students
POTENTIAL UNINTENDED ADVERSE EFFECTS				
Negative body image	Negative body perception, body weight dissatisfaction	Web-based questionnaire	Baseline, 1st follow-up and 2nd follow-up	Students
Sports injuries	Sport injuries the past 6 months	Web-based questionnaire	Baseline, 1st follow-up and 2nd follow-up	Students
Dieting	Dieting the past 6 months	Web-based questionnaire	Baseline, 1st follow-up and 2nd follow-up	Students
MODERATORS				
Gender	Boy/girl	Web-based questionnaire	Baseline, 1st follow-up and 2nd follow-up	Students
Socioeconomic position	Family occupational class, parental education	Web-based questionnaire	Baseline, 1st follow-up and 2nd follow-up	Students
IMPLEMENTATION				
Implementation measures	Dose (delivered and received), fidelity, appreciation, acceptability, context	Web-based questionnaire	1st follow-up	Students, teachers, school administrators

PA = physical activity. ^*^Baseline (August 2016), 1st follow-up (May 2017), 2nd follow-up (April 2018). ^**^ Determinants are measured for all primary and secondary outcomes. These determinants will be described in detail in future articles

A week before data collection, information materials were sent to school coordinators with separate material to each school class including unique passwords for each of the students to access an internet-based questionnaire. Students answered the questionnaire in class after a standardised instruction given by a teacher. The questionnaires can be completed within one school lesson of 45 min. Teachers were asked to encourage absent students to answer the questionnaire at a later timer. E-mails were sent to the school coordinator with an enquiry of reminding school classes of the data collections where no or few students completed the questionnaire within the timeframe.

#### School administrator questionnaires

The items for the school administrator questionnaire were developed specifically for the HHS study by the research group. The items are inspired by staff questionnaires from the DNYS [30], the HBSC study [45], the Boost study [46] and the X:IT study [48]. The questionnaire covers the following themes: Structural, physical, educational and social school environment with a specific focus on past and ongoing initiatives regarding well-being, sense of community, stress, sleep, PA and meal habits as well as organisational capacity to implement health promoting initiatives. The 20-min questionnaire was completed by the principal or school coordinator.

#### Student registration of perceived stress

Students accepted to participate in a monthly sub-study about perceived stress where text messages were used for data collection by typing their telephone number in the baseline questionnaire. The aim of this sub-study is to explore how students’ levels of stress fluctuate throughout a school year. Students received a text message the last Wednesday of each month (from September 2016 to June 2017) with the following question: 1) *Do you feel stressed? Write* e.g. *0 if your answer is ‘No, not at all’. Write only one number (0. No, not at all, 1. Yes a little, 2. Yes, a lot)*. After replying to the first message a second message was sent: 2) *How often have you felt stressed during the last month? Write* e.g. *1 if your answer is ‘A few days’. Write only one number (0. Never, 1. A few days, 2. Weekly, 3. Daily)*. If the students did not answer the questions within 24 hours, a reminder was sent automatically. An outro message was sent to all students after each data collection including contact information to a free telephone and online counselling service specifically for young people. A prior the data collection, a small pilot study was conducted to test phrasing of the text messages, guidance for answering the questions and whether the technique was working.

#### Objective assessment of PA

PA was objectively measured using Axivity AX3 accelerometers in a sub sample of two intervention schools and two control schools at baseline and first follow-up. Axivity AX3 accelerometer was attached directly to the skin of the front thigh of participating students by trained research staff. For up to seven consecutive days, the participants were instructed to wear the accelerometers all the time (including water activities and sleep). Acti 4 software will be used to discriminate between PA types including sitting, standing, walking, fast walking, running, cycling, sit-to-stand movements (i.e. transitions from sitting to upright stand), and number of steps based on threshold values of standard deviation of acceleration and the derived inclination [49].

### Primary outcome measure

The primary outcome measure is student life satisfaction at 1st follow-up measured by an adapted version of the Cantril Ladder of Life Scale for use among adolescents [50–51]. A previous study found that this version of the Cantril Ladder showed good reliability and validity among adolescents [50]. Students are asked to rate their life satisfaction using a visual analogue scale. The Cantril Ladder has 11 steps: the top indicates the best possible life and the bottom the worst. Students are asked to indicate where on the ladder they would place their lives at present (from zero to 10). **Pre-specified success indicator:** a 6%-point difference in the prevalence of students with a high level of life-satisfaction (9–10) in intervention schools compared to control schools at 9-month follow-up. This difference will be sustained at 20-month follow-up.

The 5-item WHO Well-being Index (WHO-5) [52, 53] capture the HHS projects’ focus on emotional well-being to a greater extent than Cantril Ladder. However, as no previous data on the project’s target group was available for power calculations or for estimating current response distribution on WHO-5, the Cantril Ladder was used for power calculations. WHO-5 will be included as sensitivity analyses in the primary effect evaluation.

*The WHO-5* is a short and generic global rating scale measuring subjective well-being [52]. The WHO-5 covers five positively worded items related to positive mood, vitality and general interest: ‘*I have felt cheerful and in good spirits*’, ‘*I have felt calm and relaxed*’, ‘*I have felt active and vigorous*’, ‘*I woke up feeling fresh and rested*’ and ‘*My daily life has been filled with things that interest me*’. Students are asked to indicate how well each of the five items applied to them the last 14 days. The five items are scored from 0 (none of the time) to 5 (all of the time). Theoretically, the score therefore ranges from 0 (absence of well-being) to 25 (maximal well-being). Scales measuring health-related quality of life are normally translated to a percentage scale from 0 to 100, and it is therefore recommended to multiply the WHO-5 score by four.

### Secondary outcome measures, proximal outcomes and side effects

*Secondary outcomes* are measured at all three time points in the student questionnaire and include: 1) stress measured by the 10-item Perceived Stress Scale as well as stress intensity and stress frequency, 2) sleep measured by sleep quantity and quality, 3) PA measured by hours of moderate-to-vigorous PA per week, hours of daily sedentary time and average daily PA, 4) meal habits measured by daily intake of breakfast, daily intake of lunch, daily intake of snacks and daily intake of water, and 5) sense of community within the school class and at school.

*Potential positive side effects* (such as healthy weight development, high perceived levels of energy for academic achievement, high perceived ability to concentrate during school day, decrease in school absence, decrease in intake of unhealthy food and drinks) and *unintended adverse effects* (such as negative body image, sports injuries and dieting) are measured in the student questionnaire at all three time points. *Proximal outcomes* include individual determinants (measured in the student questionnaires), interpersonal determinants (measured in the student questionnaires and in the school administrator questionnaires), and organisational determinants (measured in the school administrator questionnaires) (Table 2).

### Process evaluation

All steps of the implementation process are assessed and explored by a thorough process evaluation. A systematic process evaluation protocol developed by Aarestrup et al., 2014 [54] are used to plan the process evaluation of the implementation. Theoretically, the process evaluation of the HHS study is structured according to the conceptual frameworks presented by Linnan & Steckler [55], Rogers’ diffusion of innovation theory [56] and Durlak & Dupre [57]. The aim of the process evaluation is to 1) evaluate the recruitment of high schools (barriers and facilitators of participation) to characterise participating/non-participating schools and the appeal of the intervention to high schools, 2) collect information on the intervention dose delivered by school staff and received by students to enable analysis of the association between level of implementation and effectiveness of the intervention, 3) measure the quality of the delivered intervention, 4) examine if the intervention components reached all students irrespective of e.g. socio-demographic characteristics, 5) identify barriers and facilitators for implementation, 6) assess students’ and school staffs’ acceptability and satisfaction with the intervention, and 7) explore contextual factors of influence and interpretation of effect estimates such as competing health promoting initiatives at high schools and similar activities at control schools during the intervention year (contamination issues). The process evaluation is based on multiple data sources including questionnaires among students, teachers and school administrators, participant observations, ethnographic fieldwork at two high schools, website and app track records as well as focus group interviews and (telephone) interviews with students, teachers, school coordinators, canteen staff and student counsellors. To evaluate the implementation of MI in classrooms, students from the text message survey (see above) answered the following question in October, January and March 2016–17: *Think about last week: Did you have activity breaks during class* e.g. *brain breaks, walk & talks or other types of movement giving you a break from sitting down? Write* e.g. *4 if your answer is ‘No’. Write only one number (0. Yes, in each lesson, 2. Yes, in some lessons, 3. Yes, in very few lessons, 4. No).*

### Planned statistical analyses

The presentation of study findings will be in accordance with CONSORT guidelines for cluster-randomised controlled trials [58]. We will apply multilevel methods to analyse the effect of the intervention on the primary and secondary outcomes taking the hierarchical nature of data (students nested within classes nested within high schools) into account. We will apply an intention-to-treat approach and use multiple imputations to impute missing data as suggested by Graham (2012) [59]. To improve precision, estimates will be adjusted for baseline measurements of prognostic variables [60]. All further impacts of the intervention such as intended proximal effects (determinants), potential positive side effects and unintended adverse effects will also be subject to evaluation. We will analyse effectiveness of the intervention in different socio-demographic subgroups such as boys and girls and high and low socio-economic position. Based on the multiple pathway approach, mediation analyses will be used to examine whether changes in primary outcomes are following changes in secondary outcomes and determinants [61, 62]. The mediation analyses will be completed by Stata Software (StataCorp LP, College Station, Texas).

### Health economic evaluation

Using cost consequence analysis [63], we will calculate the total societal cost of the intervention and present this alongside different consequences of the intervention: 1) the measured changes in primary and secondary outcomes and positive and negative side effects, 2) staff’s and students’ acceptability and appreciation of the intervention, and 3) implementation challenges reported by school staff. In addition, budget impact analyses will be performed. This approach allows decision makers to evaluate and prioritise cost and consequences of implementing the HHS study in their local context [63]. The resources used for the intervention will be extracted from the project budget. The costs associated with the intervention schools’ implementation of the teaching material, initiatives of the catalogue focusing on organisational and environmental changes as well as the peer-led innovation workshop and derived activities was collected retrospectively as part of the follow-up school administrator questionnaire. Expenses include e.g. purchase of additional textbooks to support the curricular activities of the HHS study, purchase of supplies and equipment for the MI in the classroom and new after-school activities derived from the innovation workshop, salaries of additional staff required to implement the intervention (e.g. student counsellors and school coordinators) and time spent on planning implementation at the high schools.

### Power and sample size

Power calculations were performed prior to the study to assess the adequate sample size of high schools and students needed to detect an effect of the intervention between intervention and control schools in well-being. We calculated the power of a two-sided test using the *sampsi* and *sampclus* commands in STATA (STATA statistical software, version 15.0) to adjust for cluster-design effect. Conventional levels of statistical power (0.8) and level of significance (0.05) were used in the two-sided test. Data from the cross-sectional DNYS data [30] were used to estimate the average school size (number of students = 221), Intra Class Correlation (ICC) (= 0.01) and a public health relevant, realistic and detectable effect size for the primary outcome measure. The only measure of well-being in this survey was a measure of life satisfaction based on the Cantril Ladder of Life Scale [50]. This scale did not reflect the project’s concept of well-being perfectly but was the best available measure. As the 2014 prevalence of first-year students reporting high level of life satisfaction varied considerably around the mean of 22% (= median) ranging from 9 to 38%, we also aimed to move the school distribution curve of prevalence of students with high level of life satisfaction to the right. Assuming a baseline level of 22%, we aimed at a 6%-point difference in the prevalence of students with high level of life-satisfaction between intervention schools and control schools at first follow-up corresponding to the 90th percentile (28%) of the 2014 distribution. We assumed that this effect would be obtainable despite the size of the baseline level (conservative approach). Based on the assumptions that each school had on average eight first-year classes with an average of 28 students per class, the power calculations [sampsi 0.22 0.28, power (0.8)] [sampclus, obsclus (221) rho(.01)] showed that a minimum of 26 schools (13 intervention and 13 control schools) were required. We included 15 schools in each group to allow for drop-outs (corresponding to an anticipated attrition rate of 10% at the student level).

## Discussion

This paper describes the protocol for a cluster randomised controlled trial to determine the effectiveness of a school-based intervention aimed at improving well-being among high school students. The HHS study will contribute to new and important knowledge on multi-component interventions targeting multiple adolescent health behaviours simultaneously and thereby consider the potential behavioural clusters described in other studies. Furthermore, the HHS study will gain insight into challenges and potentials of working with young people in intervention research. This knowledge may comprise an important basis for evidence- and practice-based recommendations to high schools, politicians and other stakeholders. It remains a challenge to ensure sufficient implementation when intervening in schools. The thorough process evaluation of implementation processes, barriers and facilitators in the HHS study will yield important knowledge on how to improve implementation of school-based interventions. To our knowledge, no former studies have collected data about perceived stress among adolescents using text messages. The HHS study will provide answers to the applicability of this method as means of data collection among high school students. The use of text messages made it possible to repeat the data collection multiple times over a longer period and thereby explore how students’ levels of stress fluctuate throughout a school year. The HHS study also used accelerometers for assessing PA and sedentary behaviour and will examine potential barriers and facilitators of this data collection method among high school students. We collected information about the students’ Personal Identification Number (CPR-number) which is used in all national registers. This gives us unique opportunities to carry out health-related register-based research, as it is possible to combine health registers and social registers on an individual level by the CPR-number.

Strengths of the HHS study are the 1) use of the IM for a systematic theory- and evidence-based planning process of the study, 2) multi-component intervention approach combining educational as well as organisational and environmental strategies to target the multiple determinants of change at multiple levels, 3) use of a randomised controlled trial design with long follow-up, 4) large sample size of schools and students, 5) comprehensive process evaluation design based on theoretical concepts and multiple data sources, 6) objective measures of PA and sedentary behaviour and monthly measures of perceived stress collected in two sub studies, 7) measurements of determinants and potential side effects to enable investigation of the working mechanisms, and 8) use of validated outcome measures.

The HHS study has some limitations that must be considered. The HHS study is a very complex intervention with many intervention components, intervention providers and outcomes. The HHS program theory is a simplification of reality and the action of mechanisms. In reality, feedback mechanisms between different behaviours are probably present but incorporating such feedback mechanisms would make the program theory even more difficult to communicate and impossible to use in practice. Furthermore, we did not have data to calculate ICC for WHO-5 as this measure was not included in the DNYS. We, therefore, performed our power calculations using the Cantril Ladder of Life Scale. However, the Cantril Ladder is a measure of life satisfaction and does not capture the project’s dimensions of well-being related to energy for the school day identified in the need assessment.

## Data Availability

Not applicable

## Abbreviations

CPR-number: Personal Identification Number
DNYS: Danish National Youth Study 2014
HBSC: Health Behaviour in School-aged Children
HF: Higher preparatory examination
HHS: Healthy High School
HHX: Higher commercial examination
HTX: Higher technical examination
ICC: Intra-class correlation
IM: Intervention Mapping protocol
MI: Movement integration
PA: Physical activity
STX: Upper secondary school leaving examination
WHO-5: Five item World Health Organisation Well-being Index
